# Oxidative damage in the gastrocnemius predicts long-term survival in patients with peripheral artery disease

**DOI:** 10.1038/s41514-024-00147-3

**Published:** 2024-04-05

**Authors:** Panagiotis Koutakis, Hernan Hernandez, Dimitrios Miserlis, Jonathan R. Thompson, Evlampia Papoutsi, Constance J. Mietus, Gleb Haynatzki, Julian K. Kim, George P. Casale, Iraklis I. Pipinos

**Affiliations:** 1 Department of Biology, Baylor University, Waco, TX USA; 2 Department of Surgery, University of Nebraska Medical Center, Omaha, NE USA; 3 Department of Surgery and Perioperative Care, University of Texas at Austin, Austin, TX USA; 4 Department of Neurological Surgery, University of Massachusetts Medical School, Worcester, MA USA; 5 Department of Biostatistics, University of Nebraska Medical Center, Omaha, NE USA; 6 Department of Surgery and VA Research Service, VA Nebraska-Western Iowa Health Care System, Omaha, NE USA

**Keywords:** Cardiovascular diseases, Cardiovascular biology

## Abstract

Patients with peripheral artery disease (PAD) have increased mortality rates and a myopathy in their affected legs which is characterized by increased oxidative damage, reduced antioxidant enzymatic activity and defective mitochondrial bioenergetics. This study evaluated the hypothesis that increased levels of oxidative damage in gastrocnemius biopsies from patients with PAD predict long-term mortality rates. Oxidative damage was quantified as carbonyl adducts in myofibers of the gastrocnemius of PAD patients. The oxidative stress data were grouped into tertiles and the 5-year, all-cause mortality for each tertile was determined by Kaplan-Meier curves and compared by the Modified Peto test. A Cox-regression model was used to control the effects of clinical characteristics. Results were adjusted for age, sex, race, body mass index, ankle-brachial index, smoking, physical activity, and comorbidities. Of the 240 study participants, 99 died during a mean follow up of 37.8 months. Patients in the highest tertile of oxidative damage demonstrated the highest 5-year mortality rate. The mortality hazard ratios (HR) from the Cox analysis were statistically significant for oxidative damage (lowest *vs* middle tertile; HR = 6.33; *p* = 0.0001 and lowest *vs* highest; HR = 8.37; *p* < 0.0001). Survival analysis of a contemporaneous population of PAD patients identifies abundance of carbonyl adducts in myofibers of their gastrocnemius as a predictor of mortality rate independently of ankle-brachial index, disease stage and other clinical and myopathy-related covariates.

## Introduction

Lower extremity peripheral artery disease (PAD) is a prevalent atherosclerotic disease that produces progressive narrowing and occlusion of the arteries supplying the legs. PAD affects approximately 200 million people worldwide, producing a considerable public health burden^1^. PAD is associated with increased mortality even after adjusting for cardiovascular risk factors^2^ and published death rates for symptomatic PAD patients vary between 3.3% and 7.8% per year^3^. Several studies have demonstrated an association between mortality and clinical parameters identified from patient comorbidities and evaluation at presentation. Such parameters include the presence of critical limb ischemia, diabetes mellitus (DM), hypertension, renal dysfunction, and abnormal ankle-brachial index (ABI)^4–8^. Several studies have also evaluated the correlation between the level of different serum biomarkers and mortality in PAD patients. Among a large number of serum biomarkers evaluated, those that have been previously found to correlate with mortality were high-sensitivity C-reactive protein^9–11^, homocysteine^12^, urinary albumin to creatinine ratio^12^, serum amyloid A^13^, and D-dimer^13^.

More recently several groups including our own have focused on the association of the mortality of PAD patients with functional and biochemical parameters which are related to the specific pathophysiologic pathways at work in the legs of PAD patients. The primary problem in PAD is the presence of atherosclerotic blockages in the arteries supplying the legs. Blocked arteries produce a state of ischemia and ischemia/reperfusion in the affected legs which initiates oxidative stress/damage, mitochondrial dysfunction and cytokine upregulation, producing injury to all tissues including muscles, nerves, microvessels, skin, and subcutaneous tissues^14–25^. Accumulating injury in the leg leads to progressive damage to muscle structure and function which in association with exercise-induced ischemia produce the limitation in walking ability known as claudication. In more advanced stages of PAD, worsening ischemia and the continuous loss of the integrity and function of tissues in the affected leg can eventually present as tissue loss/gangrene. Injury to the skeletal muscle of the ischemic legs (myopathy of PAD) is the better explored component of this process in PAD limbs and is a fundamental element of the pathophysiology of claudication^14–18^. At the histological level the principal characteristics of the myopathy are myofiber degeneration and fibrosis of the extracellular matrix and microvessels of the affected muscle^19,26–28^. At the biochemical level the myopathy is characterized by oxidative damage, mitochondrial dysfunction, cytoskeletal disintegration and upregulation of cytokines^15–18,20,21,25,26,29–37^. Work from several laboratories including our own has demonstrated that this myopathy is closely related to leg function, daily activity, quality of life and mortality of PAD patients^38–46^. In regards to mortality, our group recently demonstrated^47^ that two basic biochemical biomarkers of PAD myopathy, protein concentration and mitochondrial content in gastrocnemius biopsies, are predictors of 5-year mortality in PAD patients. Furthermore, a study by McDermott et al. ^38^ has shown that physical measures of this myopathy including lower calf muscle density, weaker lower plantar flexion strength and weaker knee extension power were associated with increased mortality in PAD patients.

In the present work we elected to examine the levels of oxidative damage in the myofibers of the gastrocnemius of PAD patients as a predictor of 10-year mortality. Blood biomarkers of increased oxidative stress have been shown to correlate with increased mortality and poor physical performance (measured by the physical activity questionnaire and tests measuring endpoints that include exercise tolerance, upper extremity function and higher functioning tasks) in healthy aged populations^48,49^, in patients with chronic kidney disease^50–54^, in patients with septic shock^55^ and in patients with heart failure^56–58^. Among the most frequently used biomarkers of oxidative stress are carbonyl adducts. Carbonylation is a type of protein oxidation (introduction of reactive ketone or aldehyde groups in the protein) that is produced by reactive oxygen species^59,60^. Carbonyl adducts have been used largely as serum and plasma biomarkers in epidemiological studies evaluating oxidative stress as a component of the pathophysiology of human diseases and as a correlate of morbidity and mortality, because they reflect a significant component of the oxidative stress state of the body, are chemically stable and can be measured with well-established techniques^48,61,62^. At the tissue level, carbonyl adducts are present largely as protein modifications that cannot be repaired by the cell^62–64^ and because of their early formation and cumulative nature they are one of the most commonly used markers of oxidative damage in diseased organs and limbs^62^. Tissue-based carbonyls have been shown to be increased in the affected tissues in several neurodegenerative diseases^65–70^ and diseases of aging^71–74^ but have not been used to explore correlations with mortality. Our group and others have used tissue-based, protein carbonyls, in a series of studies that have shown that oxidative stress in ischemic PAD limbs is associated with the myopathy of PAD. Levels of carbonyls in the myofibers of PAD gastrocnemius are a marker reflecting the complexity of oxidative damage suffered by the proteome of the PAD muscle and have been shown to correlate with clinical stage of disease, blood flow limitation in the ischemic leg, and myopathy severity as reflected by cytoskeletal disorganization and reduction in myofiber cross-sectional area^17,18,29,30^. With this study, we evaluate the hypothesis that carbonyl levels in gastrocnemius biopsies predict the 10-year mortality rate of PAD patients.

## Results

**Fig. 1:**
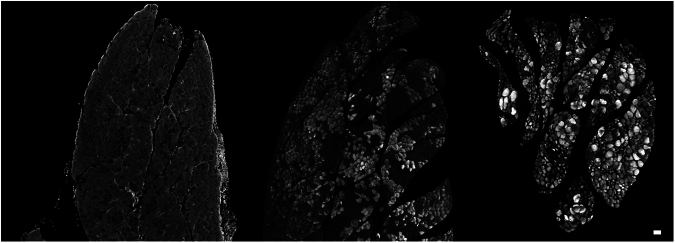
Representative images of carbonyl adducts labeling for a low, middle and high tertile gastrocnemius specimen. Multiple images were acquired (10×) and stitched together to map the myofibers on the slide. The white bar represents a length of 100 µm.

Demographic and oxidative stress descriptors, by Carbonyl tertile, are listed in Table 1. Figure 1 represents protein carbonyl images of each tertile, low (1A), middle (1B) and high (1C). The comparison among Carbonyl tertiles did not reveal statistically significant differences with respect to age (*P* = 0.598), sex (*P* = 0.130), race (*P* = 0.766), BMI (*P* = 0.175), ABI (*P* = 0.178), smoking status (*P* = 0.167), CAD (*P* = 0.500), HTN (*P* = 0.578), dyslipidemia (*P* = 0.962), family history (*P* = 0.472), renal failure status (*P* = 0.145), and statin use (*P* = 0.754). However, there were statistically significant differences among the Carbonyl tertiles with respect to survival/decedent status (*P* = 0.001), area (*P* = 0.001), DM (*P* = 0.004) and CLI (*P* = 0.001).

**Table 1:** Baseline characteristics and oxidative stress descriptors by carbonyl tertile

Variable^a^	All (*N* = 241)	Tertile 1 (*N* = 79)	Tertile 2 (*N* = 80)	Tertile 3 (*N* = 82)	*P*-value^b^
Decedents, *n*(%)	99 (41.08)	6 (7.59)	34 (42.50)	59 (71.95)	**0.001**
Age (years)	64.39 ± 9.50; 63.48 (57.84,71.50)	63.44 ± 9.82; 63.48 (56.03, 71.40)	64.51 ± 9.29; 63.46 (57.41, 71.25)	65.17 ± 9.42; 63.51 (59.72, 73.55)	0.598
Male, *n*(%)	226 (93.78)	71 (89.87)	75 (93.75)	80 (97.56)	0.130
African American, *n*(%)	16 (6.64)	5 (6.33)	4 (5)	7 (8.54)	0.766
Area	4060.85 ± 1480.48; 3961.22 (2954.00, 5108.80)	4564.57 ± 1910.10; 4715.70 (3338.19, 5922.30)	3927.97 ± 1360.01; 3907.03 (2896.66, 5007.15)	3739.28 ± 1459.58; 3758.40 (2758.00, 4793.70)	**0.001**
BMI (kg/m2)	27.36 ± 5.77; 26.97 (23.85, 31.38)	27.87 ± 5.91; 28.40 (24.54, 31.53)	27.71 ± 5.92; 26.54 (23.60, 32.09)	26.54 ± 5.47; 25.94 (23.60, 30.73)	0.175
ABI	0.44 ± 0.34; 0.38 (0.12,0.65)	0.46 ± 0.34; 0.41 (0.12, 0.66)	0.47 ± 0.33; 0.40 (0.20, 0.75)	0.40 ± 0.35; 0.30 (0.10, 0.62)	0.178
Current/Former Smoker, *n*(%)	241 (70.95)	53 (67.09)	54(67.50)	64 (78.05)	0.219
CAD, *n*(%)	88 (36.51)	26 (32.91)	28 (35.00)	34 (41.46)	0.500
HTN, *n*(%)	180 (74.69)	56 (70.89)	60 (75.00)	64 (78.05)	0.578
Dyslipidemia, *n*(%)	138 (57.26)	46 (58.23)	46 (57.50)	46 (56.10)	0.962
Family History, *n*(%)	66 (27.73)	18 (23.38)	22 (27.5)	26 (32.10)	0.472
DM, *n*(%)	101(41.91)	22 (27.85)	35 (43.75)	44 (53.66)	**0.004**
Renal Failure, *n*(%)	28 (11.62)	8 (10.13)	6 (7.50)	14 (17.07)	0.145
Statins Use, *n*(%)	145 (60.17)	50 (63.29)	46 (57.5)	49 (59.76)	0.754
Claudication, *n*(%)	126 (52.28)	54 (68.35)	41 (51.25)	31 (37.80)	**0.001**
CLI, *n*(%)	115 (47.72)	25 (31.65)	39 (48.75)	51 (62.20)	**0.001**

^a^Continuous variables summarized as mean ± SD, Median (IQR: Q1, Q3), and categorical variables as *n*(%).

^b^*P*-values from the (nonparametric) ANOVA for continuous variables, and the Chi-square test for categorical variables

*HTN* hypertension, *BMI* body mass index, *ABI* ankle-brachial index, *CAD* coronary artery disease, *DM* diabetes melitus, *Family History* family history of atherosclerotic arterial disease, *Claudication* peripheral artery disease (PAD) stage II, and *CLI* PAD Stages III/IV (Critical Limb Ischemia).

The results from the univariate Cox proportional hazards regression analyses are shown in Table 2. race and carbonyl tertile were not analyzed/shown since there were too few events in some subgroups. Area was analyzed in the univariate model as both continuous and categorized in tertiles; however, in the multivariate model it was included only as continuous to preserve the information in it. For continuous covariates, eg, age (5 units/yrs) and HR = 1.166 mean that 5 years increase in age implies 16.6% increase in hazard/risk of death. The continuous covariates Carbonyl, ABI, and age were transformed before analysis since they were not Normally distributed.

**Table 2:** Univariable predictors of mortality (Cox Model)

Risk factors	HR	95% CL^a^	*P*-value
Carbonyl (300 units)	1.631	1.392–1.910	**0.001**
Area (500 units)	0.886	0.820–0.959	**0.003**
**Area tertile**			**0.027**
Lowest	Ref.		
Middle	0.766	0.466–1.259	0.293
Highest	0.482	0.267–0.824	**0.012**
**PAD stage**			
CLI vs Claudication	1.868	1.187–2.938	**0.007**
**Obesity**			
Yes vs No	0.856	0.536–1.367	0.515
**CAD**			
Yes vs No	1.204	0.775–1.870	0.409
**HTN**			
Yes vs No	1.831	1.044–3.213	**0.036**
**Dyslipidemia**			
Yes vs No	1.265	0.809–1.977	0.303
**Family history**			
Yes vs No	0.857	0.507–1.450	0.565
ABI (0.2 units)	0.845	0.734–0.974	**0.020**
Age (yrs) (5 units)	1.166	1.042–1.305	**0.007**
BMI (kg/m^2^) (4 units)	0.871	0.749–1.013	0.073
**Smoking status**			0.247
Nonsmoker	Ref.		
Former smoker	0.579	0.305–1.102	0.096
Current smoker	0.810	0.478–1.372	0.433
**DM**			
Yes vs No	1.599	1.034–2.471	**0.035**
**Renal failure**			
Yes vs No	1.648	0.890–3.053	0.112
**Statins use**			
Yes vs No	0.796	0.513–1.235	0.309

*HTN* hypertension, *BMI* body mass index, *ABI* ankle-brachial index, *CAD* coronary artery disease, *DM* diabetes melitus, *Family History* family history of atherosclerotic arterial disease, *Claudication* peripheral artery disease (PAD) stage II, *CLI* PAD Stages III/IV (Critical Limb Ischemia).

^a^Wald Confidence Limits (CL).

The results from the multivariate Cox proportional hazards regression analyses are shown in Table 3. The effects of Carbonyl (*P* = 0.001), HTN (*P* = 0.036), age (*P* = 0.044) and smoking status (*P* = 0.005) were still statistically significant, whereas the effect of area (*P* = 0.107) was only marginally significant, and the effects of PAD stage (*P* = 0.440), ABI (P = 0.259), BMI (*P* = 0.233), and DM (*P* = 0.971) were far from statistically significant. We compared two models, one with the linear effect of Carbonyl (which needed to be modified since *P*-value = 0.007) and another with both a linear and quadratic effect of Carbonyl (which looked very reasonable, P-value = 0.325); in both models Age was with just a linear effect and behaved very well (*P*-value = 0.044).

**Table 3:** Multivariate predictors of mortality (Cox Model)

Risk factors^a^	HR	95% CL^b^	*P*-value*
Carbonyl (300 units)	1.622	1.380-1.907	**0.0001**
Area (500 units)	N/A	N/A	0.107
**PAD Stage**			
CLI vs Claudication	N/A	N/A	0.440
**HTN**			
Yes vs No	1.86	1.041-3.323	**0.036**
**Dyslipidemia**			
Yes vs No	N/A	N/A	0.199
ABI (0.2 units)	N/A	N/A	0.259
Age (yrs) (5 units)	1.136	1.003-1.287	**0.044**
BMI (kg/m^2^) (4 units)	N/A	N/A	0.2331
**Smoking Status**			
Current/former smoker vs nonsmoker	2.225	1.271-3.895	**0.005**
**DM**			
Yes vs No	N/A	N/A	0.971

*HTN* hypertension, *BMI* body mass index, *ABI* ankle-brachial index, *CAD* coronary artery disease, *DM* diabetes melitus, *Claudication* peripheral artery disease (PAD) stage II, *CLI* PAD stages III/IV (Critical Limb Ischemia).

^a^Numeric variables summarized as Mean (SD) and Median (Q1, Q3), and categorical variables as percentages.

^b^Wald Confidence Limits (CL).

*P-values from Cox model: the Wald test for numeric covariates, and the Chi-square test for categorical covariates.

From the above results, it is obvious that the effect of Carbonyl adducts is dominant, and it has subsumed even the effects of critical clinical parameters like hemodynamic severity of occlusive disease and stage of PAD and myopathic parameters like myofiber area. In particular, when the Carbonyl value increases by 300 units, the risk of death increases by 62.2%. Importantly, Harrell’s concordance index *C* = 0.74 is considerably larger than 0.5, which means that the Cox regression model has reasonable predictor power even with only a limited number of events (deaths).

**Fig. 2:**
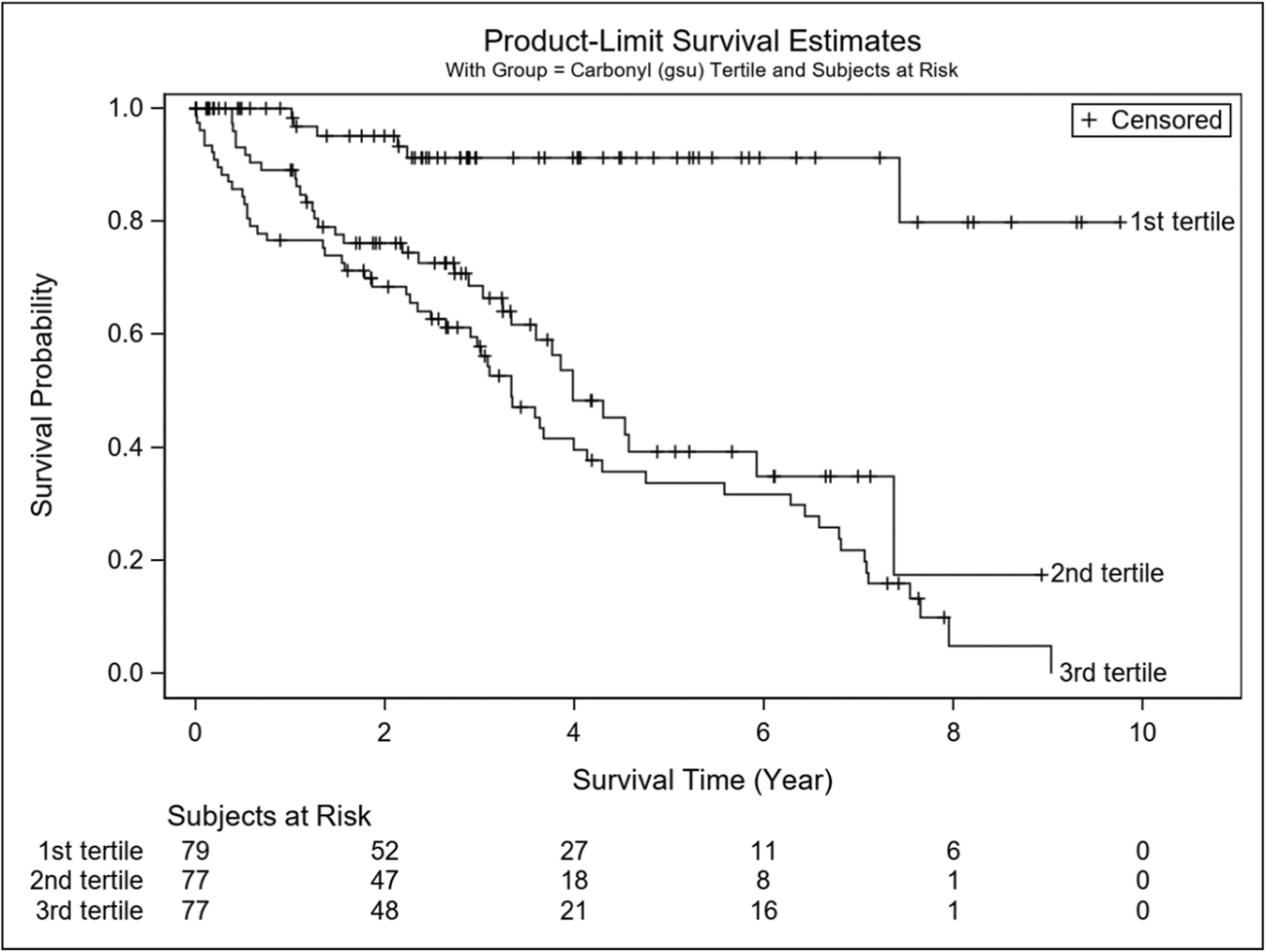
Kaplan-Meier survival curves for the tertiles of Carbonyl adducts. Tertiles were defined as low (1^st^ tertile), middle (2^nd^ tertile) and high (3^rd^ tertile) and censored events were noted (+) in the 10-year survival curves. Patients in the highest tertile of oxidative damage, quantified as carbonyl adducts in myofibers of the gastrocnemius, demonstrated the highest five-year mortality rate. GSU Carbonyl adducts were quantified as grey-scale units.

The Kaplan–Meier survival curves for the tertiles of Carbonyl adducts are shown in Fig. 2. Of note, whereas Carbonyl was used as a continuous predictor variable in all analyses (results are shown in Tables 1–3 above), for the purposes of describing survival, we categorized Carbonyl into tertiles.

Deceased and surviving PAD patients did not differ with regards to race, statin use, current tobacco use, or presence of: CAD, dyslipidemia, renal insufficiency, or family history of atherosclerotic arterial disease. Using univariate analysis (Table 2), surviving patients were of younger age (*p* = 0.009), had higher BMI (p = 0.019) and lower ABI (*p* = 0.042). Furthermore, surviving patients had lower frequency of hypertension (*p* = 0.035), Type II Diabetes Mellitus (p = 0.008) and CLI (*p* < 0.0001) and higher frequency of claudication (*p* < 0.0001), compared to decedents.

Univariate and multivariate Cox proportional hazards regression analyses were used controlling for the demographics described previously in Table 1. All predictor variables that entered our final Cox model satisfied the Proportional Hazards (PH) assumption. Carbonyl adducts were used in the Cox model in two different ways: (i) as a continuous predictor variable; and (ii) as a 3-level categorical one, by tertiles. Three parameters were predictive of mortality in our model: carbonyl adducts as a continuous predictor (HR = 1.002; *p* < 0.001), age (HR = 1.03; *p* = 0.016) and BMI (HR = 0.95; *p* = 0.014). This means that when all other factors are equal, within the 5-years period an increase of one gsu unit of carbonyl adducts in the gastrocnemius is associated with an average 0.2% increase in the HR, an increase of one year in age is associated with an average 3% increase in the HR, and a decrease of one kg/m^2^ in the BMI is associated with an average 0.05% increase in the HR. The rest of the parameters including sex, race, ABI, current tobacco use, or presence of: family history of atherosclerotic disease, CAD, dyslipidemia, DM, renal insufficiency, clinical presentation of PAD (claudication versus critical limb ischemia) and use of statin were not predictive of mortality in our cohort. When carbonyl adducts were categorized by tertiles, and used in the Cox model as categorical predictor, the hazard ratios for carbonyl adducts for the lowest (reference), middle, and highest tertiles were 1.00, 6.33, and 8.37 respectively. The middle tertile compared to the lowest tertile was significantly different at *p* = 0.0001 (Table 2). Similarly, the highest tertile was statistically different compared to the lowest tertile at *p* < 0.0001 (Table 2). Kaplan-Meier survival plot of tertiles for carbonyl adducts is presented in Fig. 1. Table 3 provides a breakdown of the lowest, middle, and highest tertile values for carbonyl adducts and the five-year mortality. Patients at the lowest tertile had the best survival. At 5 years, the mortality in this group was 8.7%. Patients in either the middle or highest tertile for carbonyl adducts in their gastrocnemius had the worst survival with 5-year mortality of 54.9% and 64.5%, respectively. The lowest tertile was statistically different from both the middle tertile (*p* = 0.001) and the highest tertile (*p* < 0.001) using the modified Peto test (Table 3). Importantly, the observed statistically significant differences persist beyond the 5-year follow-up mark with the 10-year mortality being 17.8% for patients in the first tertile, 66.9% for the patients in the second tertile and 93.1% for the patients in the third tertile. However, statistically significant conclusions concerning, e.g., the 10-year survival should be considered exploratory rather than confirmatory since the numbers-at-risk beyond the 6-year follow-up is rather small for all three tertiles and the estimates lack stability.

## Discussion

We measured protein carbonyls as a biomarker that reflects the quantity of oxidative damage suffered by the ischemic legs of PAD patients and found that the levels of carbonyl adducts in the gastrocnemius myofibers, can predict the 10-year mortality rates of patients with PAD. Our multivariate Cox regression model, demonstrated that the prognostic significance of the level of oxidative damage in the gastrocnemius was independent of severity of occlusive disease, clinical stage of PAD and other cardiovascular risk factors. Our findings suggest that needle biopsy of the gastrocnemius, and potentially, other non-invasive surrogate markers for oxidative damage in the muscle, can provide vascular specialists with a tool to stratify PAD patients at low or high risk for 5-year mortality, allowing a personalized approach (more aggressive management, intervention and follow up for higher risk patients and less aggressive for lower risk patients) to providing care for PAD patients. It is possible that gastrocnemius carbonyls can be combined with several parameters previously shown to be associated with mortality in PAD patients, to construct a high-risk profile for PAD patients. Such a profile may synthesize the information from history/physical exam (presence of diabetes, hypertension, renal dysfunction, critical limb ischemia)^5,8–10^, noninvasive hemodynamic testing (ABI)^4,75^, blood and urine tests (high-sensitivity C-reactive protein, homocysteine, urinary albumin to creatinine ratio, serum amyloid A, and D-dimer levels)^9,12,13,76,77^, muscle biopsy (carbonyl adducts, protein concentration, citrate synthase measurements)^17,18,31,47^, leg imaging (calf muscle density) and a muscle performance test (weaker lower plantar flexion strength and knee extension power)^38^. The utility of such a profile should be tested in the near future as it may be able to not only direct the general care (aggressive management, earlier intervention, more frequent follow up) for PAD patients in a personalized fashion but to also help selection and monitoring of therapy (different types of medications versus exercise therapy versus endovascular or open revascularization versus combination approach) based on the context and pathophysiological circumstances surrounding the biomarkers that are found to belong in such a profile.

The positive association between the quantity of carbonyl adducts in the gastrocnemius and the mortality observed in our study is consistent with prior data demonstrating the central importance of oxidative stress in the pathophysiology of PAD. Several groups have shown that a single bout of claudication is associated with increased oxidative stress^30,78–82^ and compromised antioxidant defenses^30,83^ within the plasma and skeletal muscle of PAD patients and animal models of hindlimb ischemia. We have also shown a significant increase in the levels of oxidative damage in PAD muscle and its myofibers compared to controls^17,18^. Oxidative damage in PAD muscle increases as the hemodynamics of the blood flow to the leg worsens and as the disease severity progresses through Fontaine stages II–IV^17^. Furthermore, oxidative damage is not a homogeneously diffuse process but varies from myofiber to myofiber with a preference to Type II myofibers, while myofibers with increased oxidative damage demonstrate reduced size and abnormal shape suggesting that oxidative damage produces progressive degeneration and necrosis of the affected muscle cells^17,18^.

The current study furthers our knowledge of the importance of oxidative damage in the pathophysiology of PAD by demonstrating a strong connection between the mortality rate of patients with PAD and the oxidative damage in the gastrocnemius of their affected legs. We offer two possible interpretations for our findings. First, increased carbonyl levels in the gastrocnemius is a reflection of a continuum of increased muscle damage, impaired leg performance, decreased physical activity, compromised general health and subsequent mortality for the PAD patient. Second, PAD produces both local damage to the tissues of the affected lower limbs (due to ischemia and ischemia/reperfusion of the leg) but also systemic effects to the rest of the body (mediated through the activation of metabolic, neural or inflammatory pathways). In this process the ischemic limb distributes and communicates to the rest of the body, the adverse ischemic events it suffers several times during the day, every time the patient experiences leg ischemia with or without associated claudication symptoms. Oxidative damage is likely a key mechanism operating to produce both the local and the systemic effects of PAD. The best evidence for a systemic effect of PAD comes from work showing that a single bout of exercise of the PAD legs produces a significant increase of biomarkers of oxidative stress (increased malondialdehyde, consumption of anti-oxidants)^83^, increased inflammatory cells (white blood cells and neutrophils)^78,84^ and increased inflammatory cytokines (thromboxane, p-selectin, von Willebrand factor) in the blood and a significant activation of the sympathetic system of PAD patients^85–87^. Of note the group of Sinoway and Cauffman have shown in several elegant studies that it takes a few, low-intensity, contractions of the posterior calf muscles of PAD patients to produce significant sympathetic system activation and adverse effects on physiologic parameters like the heart rate, blood pressure and coronary, renal artery blood flow^85–87^. It is likely that the increased local production of reactive oxygen species is a key mediator for the activation of the sympathetic loops that originate in the ischemic leg and convey the injurious information to the brain and the rest of the organs and limbs^88–90^.

The findings from the present study indicate that oxidative stress is a clinically significant biomarker that underlies the greater risk of mortality in patients with PAD. Oxidative stress can compromise biological functions either by directly damaging protein, lipids, and DNA or by providing a trigger to redox-sensitive transcription factors which set in motion pathways and responses that are damaging to the tissues^64,91^. Several studies indicate that reactive oxygen species (ROS) can cause specific protein modifications that lead to a change in the activity or function of the oxidized protein^64,91–95^.

Carbonylation is one of the major forms of such oxidative modifications. It results from the oxidation of amino acid side chains of proteins and it cannot be reversed or repaired^64,91,94,96^. Carbonyl groups are introduced within the protein primary structure by two distinct mechanisms. Direct protein carbonylation (metal ion-catalyzed oxidation) takes place with lysine, arginine, threonine and proline residues while indirect carbonylation (unsaturated aldehydes or carbohydrates interact with amino acids) takes place with lysine, arginine and cysteine residues forming advanced glycation- or lipoxidation- end products^63,97^. The consequent loss of function or structural integrity of carbonylated proteins is considered a widespread indicator of severe protein dysfunction^64,91,98^. One major source of ROS leakage is from dysfunctional mitochondria which can cause excessive oxidative damage to proteins^14^. Our group has recently showed that oxidative damage impairs vital processes in PAD patients, like the function of the nitric oxide system and its regulators^22^. Furthermore, ROS can trigger the redox-sensitive transcription nuclear factor (NF)-kB^99,100^ which is involved in the upregulation of pro-inflammatory cytokines such as tumor necrosis factor (TNF)-a, and IL-6^101,102^. The upregulation of inflammation from oxidative stress can contribute to the increased local and systemic inflammatory state of PAD^32,37^.

This is the first study to demonstrate the association between increased oxidative stress and mortality rates in patients with PAD. Previous studies have demonstrated an association between levels of oxidative stress and all-cause mortality in the elderly^48,49^. A recent epidemiological study from Europe, the ESTHER trial, identified an increased mortality rate in 2932 healthy elderly patients (median age 70 years) with increased oxidative stress as determined by derivatives of reactive oxygen metabolites and total thiol levels in serum^49^. In a different study, Semba et al. ^48^, measured serum carbonyls in 746 women aged 65 years and above. The authors found that increased serum carbonyl levels were associated with greater risk of mortality. Interestingly, 34.1% of the women who died in this cohort had peripheral artery disease compared to the 17.3% who survived.

Oxidative stress is central in the pathogenesis of several diseases including chronic renal failure and sepsis^103–106^. In patients with chronic renal failure oxidative stress is manifested as an increase in plasma protein oxidation with albumin being the major target^103^. Oxidation of albumin decreases plasma antioxidant defense and increases the risk of tissue injury induced by oxidative stress. Furthermore, patients that have chronic renal failure demonstrate increased carbonylation of urinary albumin by 71% compared to plasma albumin indicating the extensive damage caused by protein carbonyls. Increased oxidative damage to proteins and lipids from skeletal muscle has been observed in patients with uremia on haemodialysis^106^. In uremic patients muscle weakness and inability to exercise are the most common clinical symptoms^106^. Sepsis is a complex syndrome caused primarily by the release of bacterial endotoxin from gram-negative organisms. Sepsis occurs secondary to inflammatory responses, including production of tumor necrosis factor-α, which induces the release of reactive oxygen species, reinforcing the level of oxidative stress and protein damage^91^. Mortality rates of patients with sepsis range from 20% to 80% and is the major cause of death of patients undergoing intensive care^105,107,108^. Abu-Zidan et al. ^109^ demonstrated that in patients with severe sepsis carbonylation of plasma proteins was significantly increased and it was followed by a significant decrease of total body protein. While these studies present data on disease entities (renal disease, and sepsis) that are different than PAD they clearly make the link between markers of oxidative stress in the muscle with markers assessed from urine and systemic circulation and also demonstrate the potential of ROS produced in a diseased organ or limb. While these studies focus on renal disease and sepsis rather than PAD, they establish a connection between markers of oxidative stress in muscle with those assessed in urine and systemic circulation. Additionally, they highlight the potential for ROS generated in a diseased organ or limb to impact other tissues^110^ through the circulation or through the activation of the nervous system. These findings underscore the clinical importance of oxidative stress, and demonstrate the need for further research to enhance our understanding of oxidative stress mechanisms and the efficacy of their evaluations.

### Limitations

This is a descriptive study and therefore its principal limitation is that it cannot identify cause and effect linkages between gastrocnemius carbonyl adducts and mortality in PAD patients. Instead, the study demonstrated that carbonyl adducts are predictors of mortality in PAD patients, and consequently, points to the central importance of oxidative stress in the pathophysiology of PAD. Furthermore, our study population is fairly homogeneous, composed primarily of Caucasian males. This relative homogeneity may limit the generalizability of our findings to other populations. Studies of muscle from PAD limbs are crucial for our understanding of PAD pathophysiology but are difficult to be generalized mainly due to the need for obtaining a muscle biopsy. Redox proteomic analyses are warranted to further investigate protein carbonylation in the skeletal muscles and in the future, the value of surrogate markers of oxidative damage such as urinary and plasma carbonyl groups may help us understand the overall health deterioration of the patients with PAD.

Oxidative stress is a recognized component of PAD myopathy. Our data demonstrate that carbonyl adducts, a fundamental biochemical parameter of this myopathy, are predictors of mortality in PAD patients independently of ankle-brachial index, disease stage and other clinical and myopathy-related covariates. Future studies are needed to determine whether interventions such as exercise therapy, revascularization and medications with antioxidant effect can treat the oxidative damage of PAD limbs and improve the overall health and lifespan of the patient with PAD.

## Methods

### Participant Identification

This study was approved by the Institutional Review Boards of the VA Nebraska-Western Iowa Health Care System and the University of Nebraska Medical Center. Patients evaluated and diagnosed for symptomatic PAD in the vascular surgery clinic of the two institutions between December 2002 and May 2011, were identified for this study. Based on the Fontaine classification of PAD, symptomatic PAD patients present with claudication (Stage II PAD), ischemic rest pain (Stage III PAD) and tissue loss (Stage IV PAD). Frequently patients with Stage III and Stage IV PAD are grouped together in a category reported as Critical Limb Ischemia (CLI), as we have done in this study. For every patient, the diagnosis of PAD was based on medical history, physical examination, significantly decreased ABI (<0.9) and computerized or standard arteriography demonstrating significant stenoses and/or occlusions in the arteries supplying the lower extremities. A total of 241 patients with PAD were enrolled. Written informed consent was obtained from each patient who elected to participate.

### Comorbidities

After patient enrollment, patient characteristics and comorbidities were identified using a combination of patient interview and chart review. Age, sex, race, height, weight, and Body Mass Index (BMI) were recorded. Further, the presence of coronary artery disease (CAD), hypertension (HTN), dyslipidemia, diabetes mellitus (DM), tobacco use, renal dysfunction, a family history of atherosclerotic disease, and use of statins were determined for each patient. ABI was measured by standard procedures in the vascular laboratories of the respective institution. At the time of final analysis, an assessment of mortality status was performed using a combination of chart review and telephone interview with the patient or their family.

### Muscle biopsy collection

Muscle biopsy samples were obtained from the gastrocnemius of the more symptomatic limb of each patient. All muscle samples were obtained from the anteromedial aspect of the gastrocnemius belly, at a level 10 cm distal to the tibial tuberosity. The muscle samples were obtained with a 6-mm Bergstrom needle or using an open technique in those instances when the patient was having a vascular operation which involved an incision in his/her calf. The samples were placed immediately in cold methacarn. After 48 h in methacarn, the specimens were transferred to cold ethanol:H_2_O (50:50 v/v) and subsequently embedded in paraffin.

### Oxidative Stress Determination

Paraffin-embedded biopsies sectioned at 4 microns were labeled for quantification of ROS-induced oxidative damage in myofibers^17,18^. Protein carbonyls^17,18^ were measured by blocking endogenous biotin groups and then carbonyl adducts were biotinylated by treatment of slide specimens with 5 mM biocytin-hydrazide (Biocytin Hydrazide, product # 6769; Setareh Biotech, Eugene, OR, USA). After incubation for 1 h at room temperature, the slides were labeled for 1 h, with an Alexa Fluor® 488 conjugated streptavidin (10 µg/mL; Life Technologies-Molecular Probes, Grand Island, NY) secondary antibody. The conditions for all the fluorescent labels were adjusted to achieve maximal fluorescence signals required for quantitative analysis^17,18,111^. Carbonyl adducts were quantified as grey-scale units (gsu) for all the myofibers on the slide.

### Statistical analysis

Baseline continuous characteristics are presented as both mean ± SD and median (interquartile range, IQR: Q_1_, Q_3_), and categorical characteristics as counts *n* (%). Baseline characteristics were compared, one at a time, between the tertiles of the main predictor variable, gastrocnemius oxidative stress (tissue-based carbonyl adducts), using the nonparametric ANOVA (Kruskal-Wallis test) for continuous variables, and the Chi-square test for categorical variables. Survival was defined as the time from the date of muscle biopsy to the date of death. The primary outcome event was *all-cause death* (aka *all-cause mortality*). Tertiles, rather than quartiles or pentiles, were selected because the number of patients and, particularly, the number of events (deaths, ie, decedents) was not large. The categorical predictor variables Sex (M vs F) and Race (AA vs Other) were excluded from the analyses since there were too few events (ie, deaths) in most categories of these predictors. Univariate and multivariate Cox proportional hazards regression analyses were used to analyze the effect of oxidative stress (Carbonyl: carbonyl adducts) as a continuous variable on *all-cause death* while adjusting for the continuous covariates myofiber degeneration (Area: myofiber cross-sectional area), age, BMI, ankle/brachial index (ABI), and the categorical covariates PAD disease (dichotomized Fontaine stage: II vs III/IV), smoking status (nonsmoker vs former smoker vs current smoker), CAD (Y vs N), hypertension (Y vs N), dyslipidemia (Y vs N), family history of atherosclerotic arterial disease (Y vs N), DM (Y vs N), renal dysfunction (Y vs N), and statins use (Y vs N). The effect of continuous covariates was calculated per certain number of units, eg, per 5 units/years for Age, chosen to be approximately one decile, based on the approximation formula Range/10 = (max − min)/10. That units change did not change the respective P-values, but the values of the hazard ratios (HRs) and their confidence intervals (CIs) became better interpretable. Kaplan-Meier curves were used to visualize survival trajectories for different groups, whereas group comparisons for survival were completed using the Modified Peto test. For the continuous covariates in the Cox Proportional Hazards (PH) model, additive changes in the covariates are assumed to have constant multiplicative effects on the hazard rate, expressed as the hazard ratio HR = *exp* (beta*(x2 – x1)). In other words, each single unit change in the continuous covariate, no matter at what the base level x1 of that covariate, is associated with the same constant percent change in the hazard rate, determining a constant functional form of the hazard ratio. Cumulative martingale residuals were used to assess the proportional hazards (PH) assumption for each covariate as well as the functional form of continuous predictors in the Cox regression model^112^. When a covariate did not meet the PH assumption, an interaction term with that covariate and the time variable was added to the regression model^113^. Only covariates passing the threshold of P-value < 0.20 were included in the initial multi-variable Cox regression model. Harrell’s concordance index (*C*) was used to evaluate the goodness-of-fit of the final Cox regression model^114^. Harrell’s *C* is an easy-to-interpret coefficient since a value of *C* = 0.5 corresponds to a non-informative prediction model, whereas *C* = 1 corresponds to perfect association^115^. In biomedical applications, *C* often ranges between the values 0.6 and 0.75. All statistical analyses were performed using the statistical software SAS version 9.4 (SAS Inst. Inc., Cary, North Carolina).

### Reporting summary

Further information on research design is available in the Nature Research Reporting Summary linked to this article.

### Supplementary information

Reporting Summary

## Data Availability

The datasets used or analyzed during the current study are available from the corresponding authors upon reasonable request.

## Code availability

The analysis code is available om the corresponding authors upon reasonable request.
