# Supplementary material for: Oxidative damage in the gastrocnemius predicts long-term survival in patients with peripheral artery disease
Source: NPJ Aging. 2024 Apr 5;10(1):21. doi: 10.1038/s41514-024-00147-3 (PMC10997596; doi:10.1038/s41514-024-00147-3)
Supplement: Supplementary file 1 — Reporting Summary [file 41514_2024_147_MOESM1_ESM.pdf]

Corresponding author(s): Pangiotis Koutakis
Last updated by author(s): YYYY-MM-DD

Reporting Summary

Nature Portfolio wishes to improve the reproducibility of the work that we publish. This form provides structure for consistency and transparency in reporting. For further information on Nature Portfolio policies, see our Editorial Policies and the Editorial Policy Checklist.

Statistics

For all statistical analyses, confirm that the following items are present in the figure legend, table legend, main text, or Methods section.

n/a	Confirmed	
☐	☒	The exact sample size (*n*) for each experimental group/condition, given as a discrete number and unit of measurement
☐	☒	A statement on whether measurements were taken from distinct samples or whether the same sample was measured repeatedly
☐	☒	The statistical test(s) used AND whether they are one- or two-sided<br>*Only common tests should be described solely by name; describe more complex techniques in the Methods section.*
☐	☒	A description of all covariates tested
☐	☒	A description of any assumptions or corrections, such as tests of normality and adjustment for multiple comparisons
☐	☒	A full description of the statistical parameters including central tendency (e.g. means) or other basic estimates (e.g. regression coefficient) AND variation (e.g. standard deviation) or associated estimates of uncertainty (e.g. confidence intervals)
☐	☒	For null hypothesis testing, the test statistic (e.g. *F*, *t*, *r*) with confidence intervals, effect sizes, degrees of freedom and *P* value noted<br>*Give P values as exact values whenever suitable.*
☒	☐	For Bayesian analysis, information on the choice of priors and Markov chain Monte Carlo settings
☐	☒	For hierarchical and complex designs, identification of the appropriate level for tests and full reporting of outcomes
☒	☐	Estimates of effect sizes (e.g. Cohen's *d*, Pearson's *r*), indicating how they were calculated

Our web collection on statistics for biologists contains articles on many of the points above.

Software and code

Policy information about availability of computer code

Data collection	We used the appropriate coding as mentioned in the SAS 9.4 version software.
Data analysis	SAS version 9.4

For manuscripts utilizing custom algorithms or software that are central to the research but not yet described in published literature, software must be made available to editors and reviewers. We strongly encourage code deposition in a community repository (e.g. GitHub). See the Nature Portfolio guidelines for submitting code & software for further information.

Data

Policy information about availability of data

All manuscripts must include a data availability statement. This statement should provide the following information, where applicable:

- Accession codes, unique identifiers, or web links for publicly available datasets
- A description of any restrictions on data availability
- For clinical datasets or third party data, please ensure that the statement adheres to our policy

The datasets used or analyzed during the current study are available from the corresponding authors upon reasonable request.

Research involving human participants, their data, or biological material

Policy information about studies with human participants or human data. See also policy information about sex, gender (identity/presentation), and sexual orientation and race, ethnicity and racism.

Reporting on sex and gender	Sex was considered as a covariate in our statistical design. However, it was not significant and did not influence the data analysis.
Reporting on race, ethnicity, or other socially relevant groupings	Race and ethnicity were determined from the medical records of the patients.
Population characteristics	This study was approved by the Institutional Review Boards of the VA Nebraska-Western Iowa Health Care System and the University of Nebraska Medical Center. Patients evaluated and diagnosed for symptomatic peripheral artery disease in the vascular surgery clinic of the two institutions between December 2002 and May 2011, were identified for this study.. Based on the Fontaine classification of PAD, symptomatic PAD patients present with claudication (Stage II PAD), ischemic rest pain (Stage III PAD) and tissue loss (Stage IV PAD). Frequently patients with Stage III and Stage IV PAD are grouped together in a category reported as Critical Limb Ischemia (CLI), as we have done in this study. For every patient, the diagnosis of PAD was based on medical history, physical examination, significantly decreased ABI (<0.9) and computerized or standard arteriography demonstrating significant stenoses and/or occlusions in the arteries supplying the lower extremities. A total of 241 patients with PAD were enrolled. Written informed consent was obtained from each patient who elected to participate.
Recruitment	see above
Ethics oversight	This study was approved by the Institutional Review Boards of the VA Nebraska-Western Iowa Health Care System and the University of Nebraska Medical Center.

Note that full information on the approval of the study protocol must also be provided in the manuscript.

Field-specific reporting

Please select the one below that is the best fit for your research. If you are not sure, read the appropriate sections before making your selection.

☒ Life sciences ☐ Behavioural & social sciences ☐ Ecological, evolutionary & environmental sciences

For a reference copy of the document with all sections, see nature.com/documents/nr-reporting-summary-flat.pdf

Life sciences study design

All studies must disclose on these points even when the disclosure is negative.

Sample size	Power analysis determined the sample size.
Data exclusions	N/A
Replication	All measurements for carbonyl adducts were done in triplicate.
Randomization	Patients were categorized based on their disease stage. From Stage II to Stage III and Stage IV.
Blinding	All the investigators were blindly analyzed the data without knowing which Stage derived were the samples.

Reporting for specific materials, systems and methods

We require information from authors about some types of materials, experimental systems and methods used in many studies. Here, indicate whether each material, system or method listed is relevant to your study. If you are not sure if a list item applies to your research, read the appropriate section before selecting a response.

Materials & experimental systems

n/a	Involved in the study
☐	☒ Antibodies
☒	☐ Eukaryotic cell lines
☒	☐ Palaeontology and archaeology
☒	☐ Animals and other organisms
☒	☐ Clinical data
☒	☐ Dual use research of concern
☒	☐ Plants

Methods

n/a	Involved in the study
☒	☐ ChIP-seq
☒	☐ Flow cytometry
☒	☐ MRI-based neuroimaging

Antibodies

Antibodies used

Biocytin Hydrazide, product # 6769; Setareh Biotech, Eugene, OR, USA, Alexa Fluor® 488 conjugated streptavidin,; Life Technologies-Molecular Probes, Grand Island, NY

Validation

Biocytin hydrazide is an aldehyde-fixable cellular tracer that can be introduced into cells by microinjection and labeling of proteins, glycoproteins and carbohydrate. We validated the carbonyl labeling with reverse phase protein assay for carbonyl groups.

Plants

Seed stocks

Report on the source of all seed stocks or other plant material used. If applicable, state the seed stock centre and catalogue number. If plant specimens were collected from the field, describe the collection location, date and sampling procedures.

Novel plant genotypes

Describe the methods by which all novel plant genotypes were produced. This includes those generated by transgenic approaches, gene editing, chemical/radiation-based mutagenesis and hybridization. For transgenic lines, describe the transformation method, the number of independent lines analyzed and the generation upon which experiments were performed. For gene-edited lines, describe the editor used, the endogenous sequence targeted for editing, the targeting guide RNA sequence (if applicable) and how the editor was applied.

Authentication

Describe any authentication procedures for each seed stock used or novel genotype generated. Describe any experiments used to assess the effect of a mutation and, where applicable, how potential secondary effects (e.g. second site T-DNA insertions, mosiacism, off-target gene editing) were examined.
